# Circadian Control of Inflammasome Pathways: Implications for Circadian Medicine

**DOI:** 10.3389/fimmu.2020.01630

**Published:** 2020-07-31

**Authors:** Benoit Pourcet, Hélène Duez

**Affiliations:** University of Lille, Inserm, CHU Lille, Institut Pasteur de Lille, U1011-EGID, Lille, France

**Keywords:** clock, inflammasome, NLRP3, Rev-erb, RORalpha, circadian immunity

## Abstract

The innate immune system senses “non-self” molecules derived from pathogens (PAMPs) as well as endogenous damage-associated molecular patterns (DAMPs) and promotes sterile inflammation that is necessary for injury resolution, tissue repair/regeneration, and homeostasis. The NOD-, LRR- and pyrin domain containing protein 3 (NLRP3) is an innate immune signaling complex whose assembly and activation can be triggered by various signals ranging from microbial molecules to ATP or the abnormal accumulation of crystals, thus leading to IL-1β and IL-18 maturation and secretion. Deregulation of the NLRP3 signaling cascade is associated with numerous inflammatory and metabolic diseases including rheumatoid arthritis, gout, atherosclerosis or type 2 diabetes. Interestingly, the circadian clock controls numerous inflammatory processes while clock disruption leads to or exacerbates inflammation. Recently, the biological clock was demonstrated to control NLRP3 expression and activation, thereby controlling IL-1β and IL-18 secretion in diverse tissues and immune cells, particularly macrophages. Circadian oscillations of NLRP3 signaling is lost in models of clock disruption, contributing to the development of peritonitis, hepatitis, or colitis. Sterile inflammation is also an important driver of atherosclerosis, and targeting the production of IL-1β has proven to be a promising approach for atherosclerosis management in humans. Interestingly, the extent of injury after fulminant hepatitis or myocardial infarction is time-of-day dependent under the control of the clock, and chronotherapy represents a promising approach for the management of pathologies involving deregulation of NLRP3 signaling.

## Introduction

Organisms evolved in presence of a recurring daily light–dark cycle generated by the rotation of the Earth. To adapt to this predictable environmental change, they developed an internal clock mechanism that is entrained to and anticipates environmental cues such as light or food availability and optimizes physiological functions by ascribing them to the best time window (1). Many, if not all, physiological pathways and functions are regulated in a daily manner including sleep/active alternance, metabolism, heart rate, brain and muscular activity, to cite a few. More recently, research efforts have been focused on the circadian behavior of the immune system that allows optimization of immune responses throughout the day/night cycle (2), leading to the emerging concept of circadian immunity. As a consequence, alteration of the circadian clock aggravates acute and chronic inflammatory diseases, pointing to new pharmacological approaches (3, 4).

The NLRP3 inflammasome was identified as a critical immune component that orchestrates host immune homeostasis. However, its chronic activation by endogenous danger signals derived from tissue damage and abnormal accumulation of self-components including urea and hydroxyapatite crystals in joints, amyloid fibers in brain or cholesterol crystals in the vascular wall, contributes to the development of a wide variety of diseases (5). Hence, a tight control of its transcription and activation is required to avoid overt deleterious activation.

In this review, we summarize the current knowledge on clock-controlled inflammasome modulation and highlight the underlying mechanisms as well as gaps of knowledge. We discuss several pathological contexts in which clock alteration contributes to NLRP3-driven pathologies and the potential of a (re-) synchronization of the clock to fine-tune NLRP3 activation and restore tissue homeostasis.

## Innate Immune System and Pattern Recognition Receptors (PRRs)

The innate immune system is the first line of defense involved in the clearing of invaders like bacteria and viruses and also of abnormal accumulation of self-components including cellular debris or crystals. Immune cells discriminate infectious agents-derived molecules called pathogen-associated molecular patterns (PAMPs) and non-infectious, endogenous “danger molecules” or DAMPS (damage-associated molecular patterns) released by damaged or dying cells following tissue injury. These motifs are specifically recognized by tissue-resident cells such as mast cells, monocytes/macrophages, neutrophils and dendritic cells that express Pattern Recognition Receptors (PRRs). PRRs may be classified depending on their nature, their ligands and their cellular localization [see (6) for review]. Hence, they can be distinguished according to whether they are located at the cytoplasmic membrane (membrane PRRs: Toll-Like Receptors TLRs, C-type lectin receptors CLRs) or in the cytoplasm (cytoplasmic PRRs: NOD-Like Receptors NLRs, RIG-I-like Receptors RLRs and cytosolic DNA sensors CDSs). For instance, TLR-2 and TLR-4 are membrane receptors that are bound by PAMPs such as Gram+ peptidoglycans or Gram- LPS, respectively. Detection of PAMPs by PRRs triggers maturation and activation of immune cells that, in turn, secrete inflammatory factors and stimulate adaptive immunity (7). Non-infectious DAMPs are also recognized by PRRs on innate immune cells and initiate a so-called sterile inflammation. In addition to classical PRRs, numbers of non-PRR transmembrane proteins including Receptor for Advanced glycation endproducts (RAGEs), Triggering Receptors Expressed on Myeloid cells (TREMs), G Protein-Coupled Receptors (GPCRs) and ion channels are able to sense DAMPs and to trigger migration and activation of immune cells (6). PRRs and non-PRRs are involved in sterile inflammation and inflammatory diseases such as ischaemia-reperfusion injury, systemic lupus erythematosus, gout, neurodegenerative diseases, diabetes, colitis, atherosclerosis, hepatitis, rheumatoid arthritis, cancer, lung diseases, and gut diseases (6).

Inflammation is characterized by the production of histamine, cytokines, chemokines, and lipid derivatives (6). Cytokines are immunomodulatory signaling molecules playing a pivotal role in inflammation. The IL-1 cytokine family is composed of several members including IL-1α, IL-1β, IL-18, IL-33, IL-36α, IL-36β, and IL-36γ (7). Except for IL-1α, IL-1 cytokines are produced as inactive pro-cytokines and require maturation to biologically active forms by enzymatic cleavage. Among those, IL-1β is probably the most studied IL-1 family member because of its central involvement in acute and chronic inflammatory diseases. Pro-IL-1β, the inactive form of IL-1β, is processed by the proteolytic activity of Caspase 1, the predominant IL-1 processing protease. Caspase 1 activity is tightly controlled by cytosolic PRR-constituted inflammasome complex.

NOD-like receptors form the main class of cytosolic PRRs that are activated by diverse exogenous signals including anthrax lethal toxin (NLRP1), bacterial flagellin (NLRC4), double-stranded DNA Absent in Melanoma 2 (AIM2), Toxin-induced modifications of Rho-GTPase (Pyrin). In this regard, NLRP3 is unique because it acts as an intracellular innate immune sensor for a large variety of PAMPS and also DAMPs.

## The NLRP3 Inflammasome: A Stress Sensor

The nucleotide-binding domain (NOD)-, Leucine-rich repeat (LRR)- and pyrin domain-containing protein 3 NLRP3 inflammasome was first identified in Cryopyrin-associated periodic syndrome (CAPS) before its implication was recognized in many inflammatory/immune diseases such as gout, atherosclerosis, type 2 diabetes (T2D) and non-alcoholic fatty liver disease (NAFLD) (8), as well as neurodegenerative diseases (Alzheimer and Parkinson diseases) and aging (9–11), and infection by various pathogens (12). The NLRP3 inflammasome is mainly expressed by monocytes/macrophages, neutrophils and dendritic cells, but also by other cell types including hepatocytes (13), neurons (14), cardiomyocytes (15), pancreatic beta cells (16), or endothelial cells (17).

### A Two-Step Activation Process

The NLRP3 inflammasome is a macromolecular protein complex whose assembly is hierarchically organized and mostly requires a sensor protein, an adapter protein and an effector protein. The NLRP3 protein is a sensor protein that is composed of a C-terminal leucine rich repeat (LRR) domain, a central oligomerization domain (NOD, nucleotide-binding and oligomerization domain, NACHT) and an N-terminal Pyrin effector domain (PYD). This last PYD interacts with the amino-terminal PYD domain of the apoptosis-associated speck-like protein containing a Caspase recruitment domain (ASC) protein to initiate the inflammasome assembly and the formation of the so-called ASC speck. ASC is playing the role of adapter platform for the Caspase 1 protein thanks to its a carboxy terminal CARD domain that eventually recruits an unprocessed pro-caspase1 (18). Pro-caspase-1 oligomerization on the ASC filament enables proximity-driven autocatalytic caspase-1 maturation.

**Figure 1:**
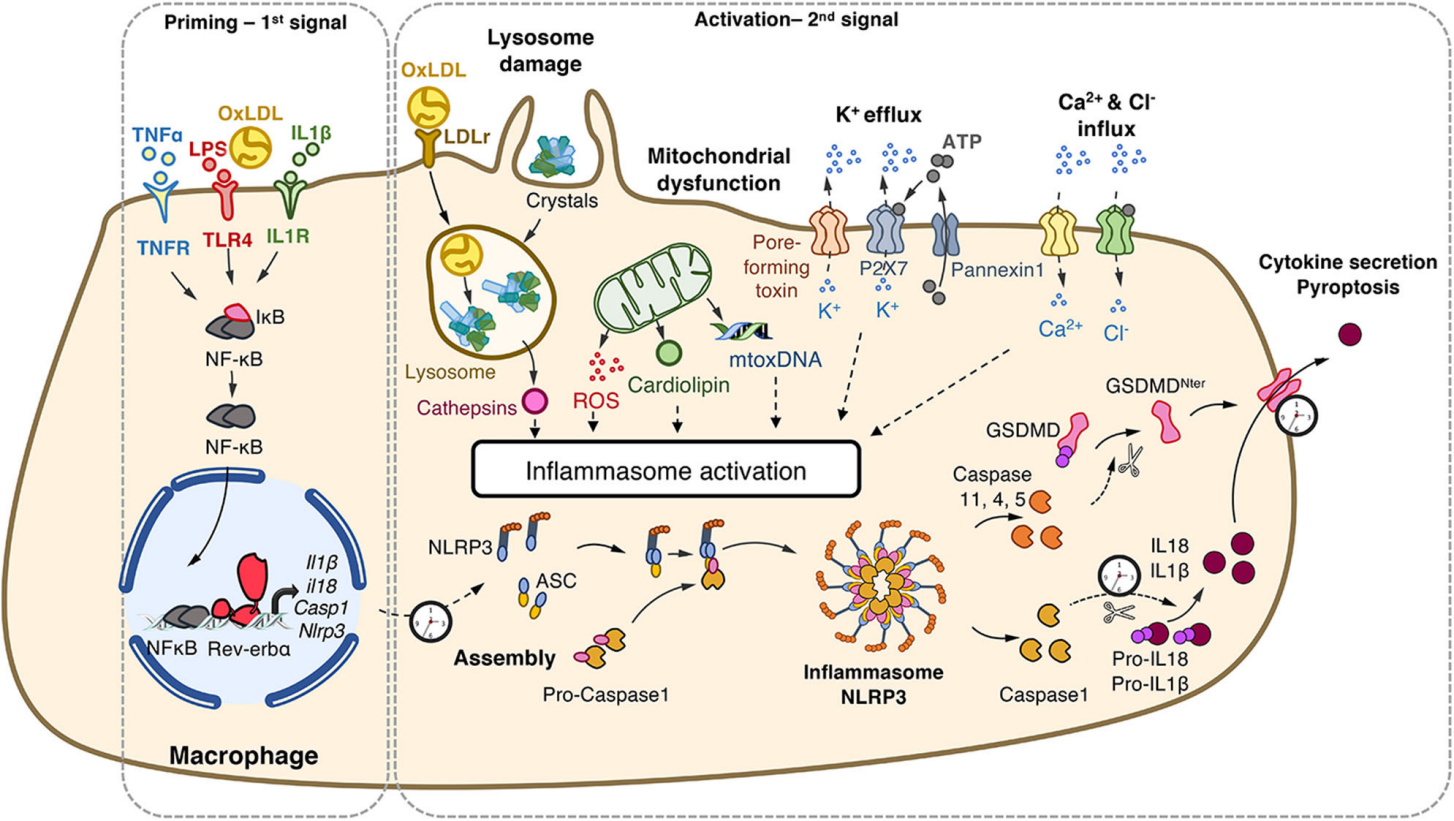
NLRP3 inflammasome priming and activation. The priming (first step) of the NLRP3 inflammasome requires the binding and activation of PRRs (TLRs,.) by PAMPs such as LPS, cytokines or ox-LDL, resulting in the transcription of the NLRP3 inflammasome components. Its activation (second step) is the result of recognition of PAMPs (such as the bacterial pore-forming toxin nigericin) or DAMPs which are released by damaged or dying cells (such as ATP) following injury or metabolic imbalance (such as mtROS), or accumulate in tissues (such as crystals). These lead to lysosomal damage, mitochondrial damages (exposition of cardiolipin, mtDNA) which ultimately modify ion (K+, Ca2+) fluxes. Upon these two-step process, the NLRP3 inflammasome assembles, caspase-1 is activated, Gasdermin-D and pro-IL-1β and pro-IL-18 are cleaved, leading to mature cytokines secretion and cell death by pyroptosis. ASC, apoptosis-associated speck-like protein containing a CARD domain; ATP, adenosine triphosphate; casp, caspase; DAMPs, damage-associated molecular patterns; GSDMD, gasdermin-D; IL, interleukin; IL1R, interleukin-1 receptor; LDL, Low Density Lipoprotein; LDLr; LDL receptor; LPS, lipopolysaccharide; mtoxDNA, mitochondrial oxidized DNA; NFκB, nuclear factor-kappa B; NLRP3, nucleotide-binding, LRR and PYD domains-containing protein 3; Ox-LDL, oxidized low-density lipoproteins; PAMPs, pathogen-associated molecular patterns; PRRs, Pattern Recognition Receptors; ROS, reactive oxygen species; P2X7R, purinergic receptor P2X 7; TLR, Toll-like receptor; TNF, tumor necrosis factor; TNFR, tumor necrosis factor receptor.

This complex activation is tightly controlled by a two-step process (Figure 1). A priming step is required to increase gene and protein expression of its components in order to sense stimuli and become activated (19). This priming occurs *via* ligand binding to PRRs (*eg*. signals that engage TLRs). These ligands may originate from exogenous sources such as bacterial wall components (Lipopolysaccharides, proteoglycans), or endogenous molecules (oxidized low-density lipoproteins [oxLDL], IL1, TNFα). This priming step is tightly controlled at the transcriptional level by the classical pro-inflammatory NF-κB and AP1 pathways, but also by metabolic sensors such as nuclear receptors including Liver X Receptors (20) and Rev-erb (21). A second step is the activation of the NLRP3 inflammasome in a primed cell/tissue that triggers the NLRP3 multimeric complex assembly that allows caspase 1 maturation and results in caspase1-mediated maturation of the pro-inflammatory interleukin-1β (IL-1β) and IL-18, the release of the mature cytokines, as well as in the so-called pyroptotic cell death (22). This second step may be triggered by a variety of compounds identifying NLRP3 as a wide PAMPs and DAMPs sensor as described below (8, 22, 23).

### NLRP3: A PAMPs' and DAMPs' Sensor

The NLRP3 inflammasome detects a broad range of DAMPs and PAMPs. Cholesterol crystals that accumulate in the arterial wall during atherosclerosis (24), monosodium urate (MSU) accumulation in joints leading to gout (25) and hydroxyapatite crystals triggering rheumatoid arthritis (5) all activate the NLRP3 inflammasome (Figure 1). The internalization of crystals leads to lysosomal damage and subsequent cathepsins and Ca^2+^ release that activates NLRP3 in a yet unknown manner. In addition, NLRP3 activation is also triggered by metabolic stresses such as hyperglycemia, some fatty acids and ceramides, and mitochondrial dysfunction, in particular mtROS (26), exposition of cardiolipin (27) or presence of mitochondrial oxidized DNA (28). Bacterial pore-forming toxins such as nigericin act as ionophores promoting K^+^ efflux which provokes the assembly of the NLRP3 complex, activation of Caspase 1 and the release of mature cytokines (29). Extracellular ATP released by dying cells also results in K^+^ and Ca^2+^ fluxes through P2X7 channel opening (23). In the same line, Ca^2+^ influx into the cytoplasm after mitochondrial reactive oxygen species (mtROS)-mediated cation channel transient receptor potential melastatin 2 (TRPM2) opening has been suggested to trigger the NLRP3 inflammasome assembly and IL-1β production in MSU-stimulated macrophages (28). The NLRP3 inflammasome is also sensing accumulation of aggregates (e.g., β-amyloid, Aβ) as well as metabolic stresses (8). Thus, the NLRP3 inflammasome is considered as a stress sensor that detects loss of homeostasis and abnormal endogenous molecules that signal infection, metabolic abnormalities or tissue damage (23).

## Circadian Control of the Immune System

### Molecular Organization of the Mammalian Clock

**Figure 2:**
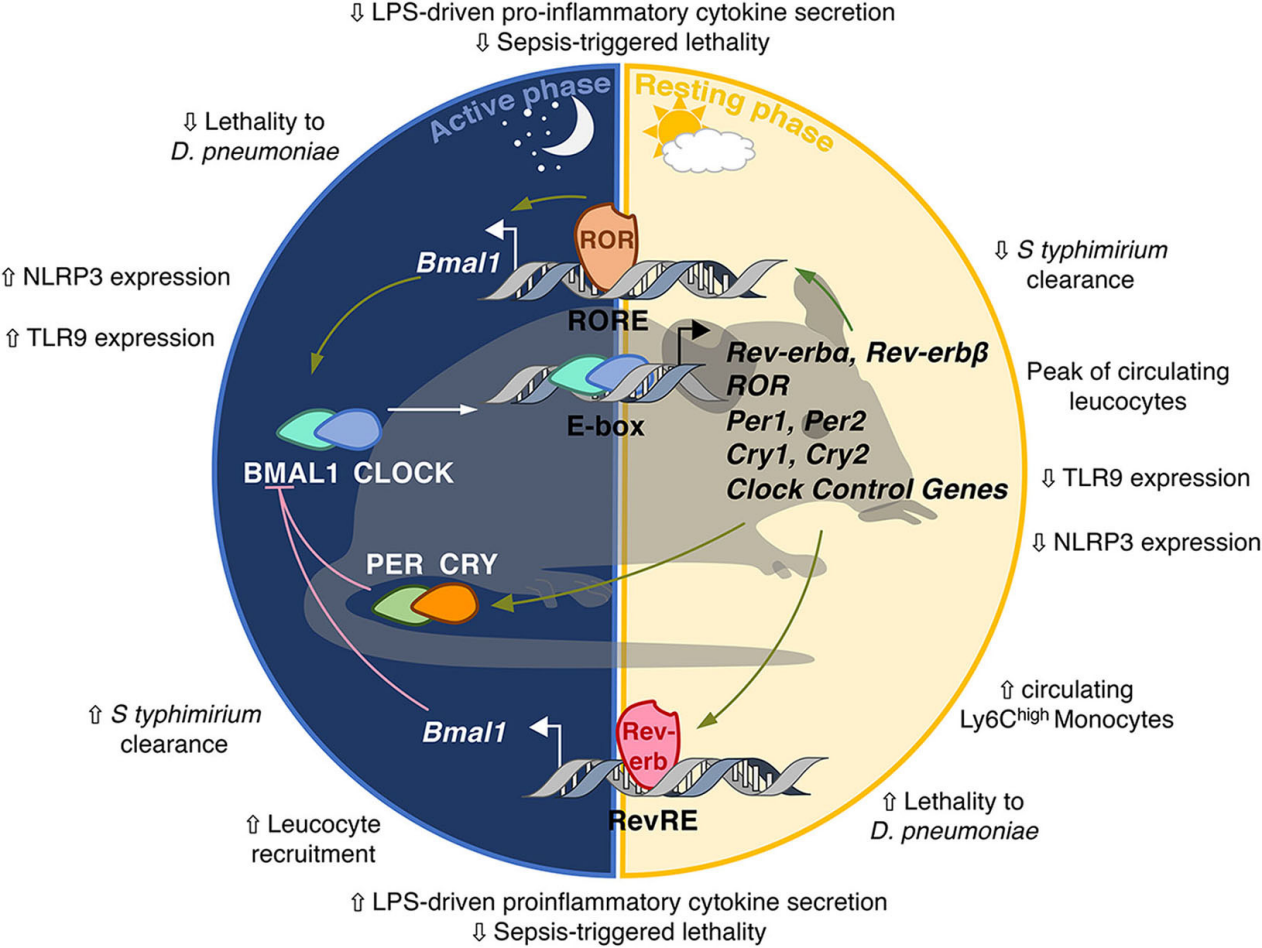
The molecular circadian clock in mammals. The molecular clockwork is formed by transcription–translation feedback loops. The transcription factors BMAL1/CLOCK induce the expression of E-box-containing genes including the negative regulators Period (PER) and Cryptochrome (CRY). In turn, the PER/CRY heterodimer inhibits the transcriptional activity of BMAL1/CLOCK. Once PER and CRY levels are sufficiently low, a new cycle may start. CLOCK/BMAL1 induce the expression of the nuclear receptors Rev-erbα/β and retinoid-related orphan receptor α, β, and γ (RORα/β/γ). Rev-erbs and RORs interact with co-repressors (NCoR) and co-activators (NCoA) and compete for the binding of RevRE/RORE elements in common target genes to repress or activate, respectively, their transcription. Rev-erb and ROR are also able to repress E4BP4/Nfil3 which rhythmically inhibits D-box-dependent transcription. Additional layers of regulation of circadian gene expression include rhythmic histone modifications, circadian chromosomal 3D conformation and post-translational modifications such as acetylation, phosphorylation, sumoylation, O-GlcNacylation.The clock is involved in the control of so-called circadian immunity.

The mammalian clock consists of transcriptional activators and repressors forming interlocked feedback regulatory loops and organized in positive and negative limbs that confer rhythmicity to each other (30) (Figure 2). The positive limb is driven by BMAL1 (Brain and Muscle ARNT-like 1) and CLOCK (Circadian Locomotor Output Cycles Kaput) which heterodimerize and bind to E-boxes in their target gene promoters, amongst which *Per* and *Cry* clock genes whose transcription is activated by BMAL1/CLOCK. Period (PER) 1/2/3 and Cryptochrome (CRY) 1/2 form the negative limb. PER and CRY, once they reach sufficient quantity in the cytoplasm, heterodimerize and translocate to the nucleus where they bind BMAL1-CLOCK heterodimers to inhibit BMAL1/CLOCK transcriptional activity in a rhythmic manner (30). This first circuitry is finely tuned by the nuclear receptors Rev-erbs and RORs (31), which compete for binding to the same RevRE/RORE and RevDR2 DNA sequences and regulate gene expression in an opposite manner. While Rev-erbs act as transcriptional repressors, RORs compete with Rev-erbs for DNA binding and activate transcription of common target genes, including *Bmal1* (32). Because Rev-erb isotypes display strong circadian rhythmicity in their abundance, this competition for binding to the *Bmal1* promoter is rhythmic and contributes to BMAL1 oscillations. It is noteworthy that these transcription factors not only control each other's transcription but also bind to numerous genes containing RORE/RevDR2 or E-boxes, thereby generating rhythmic transcriptional waves in transcriptional programs involved in local tissue functions. For instance, Rev-erb-α controls the expression of *E4bp4*/*Nfil3* in the liver (33, 34) but also in immune cells thereby regulating Th17 immune cell differentiation (35). Rev-erb also represses *Cry1* transcription thus controlling both limbs of the clock in a coordinated manner (36).

The rhythmicity observed in gene transcription is not only due to cyclic binding of these transcription factors but also to circadian variations in histone marks and chromatin organization at regulatory regions (30, 37). Beside epigenetic control, dynamic 3D chromatin architecture is another layer of circadian genome function (38). Additionally, post-translational modifications such as phosphorylation, SUMOylation, O-Glc-Nacylation are necessary to ensure the stability of these transcription factors and thus the pace and robustness of the clockwork [(39) for review].

### Biological Clocks in the Immune System

**Figure 3:**
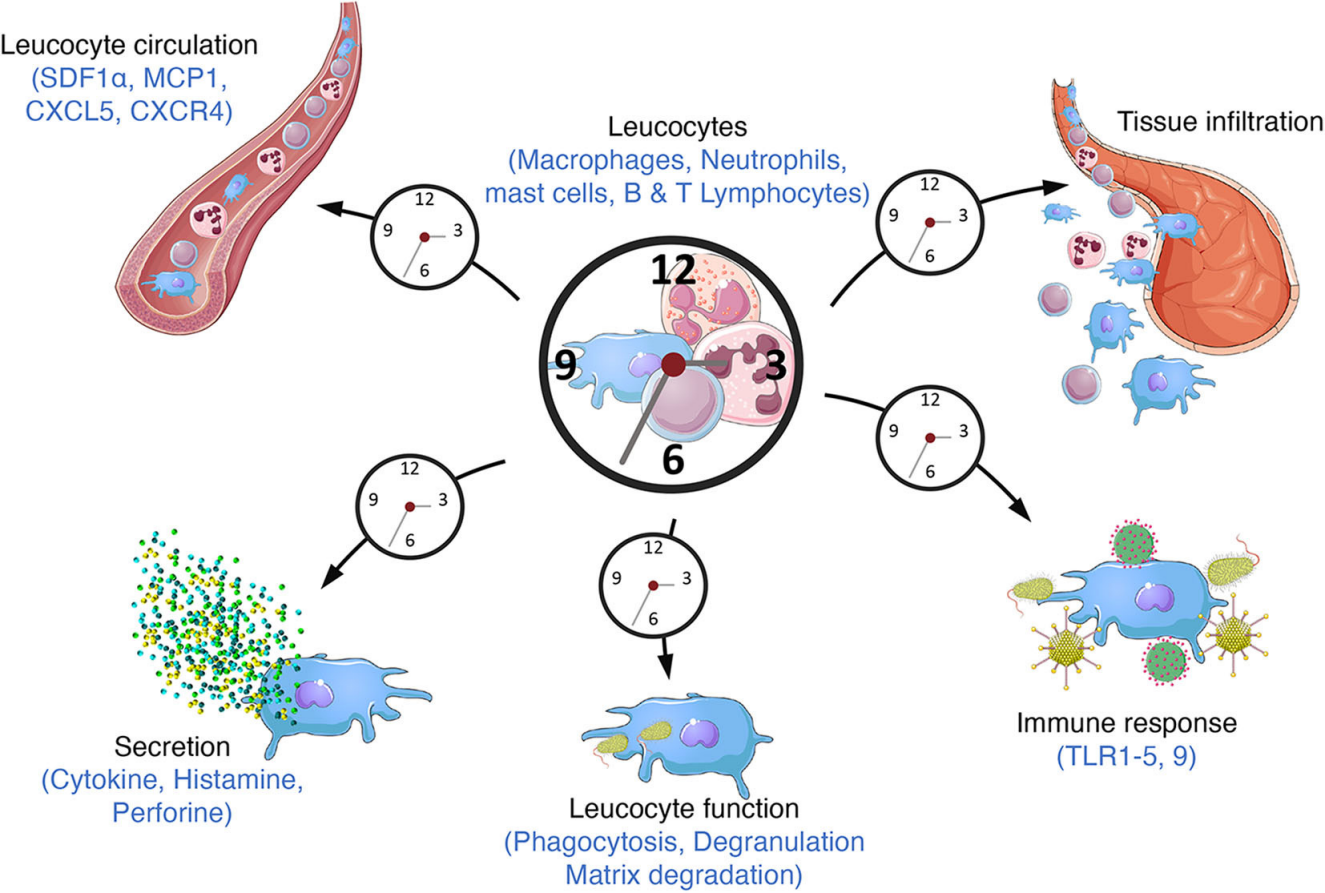
Circadian immunity. The molecular clock regulates a large number of immune functions throughout the day/night cycle such as cytokine secretion, phagocytosis, response to pathogens (bacterium, parasite) through the expression of TLR9 or the TLR4 pathway and danger signals through the regulation of the NLRP3 inflammasome. Circulating leucocyte number also varies along the day altogether with their mobilization from bone marrow and their recruitment into tissues.

**Figure 4:**
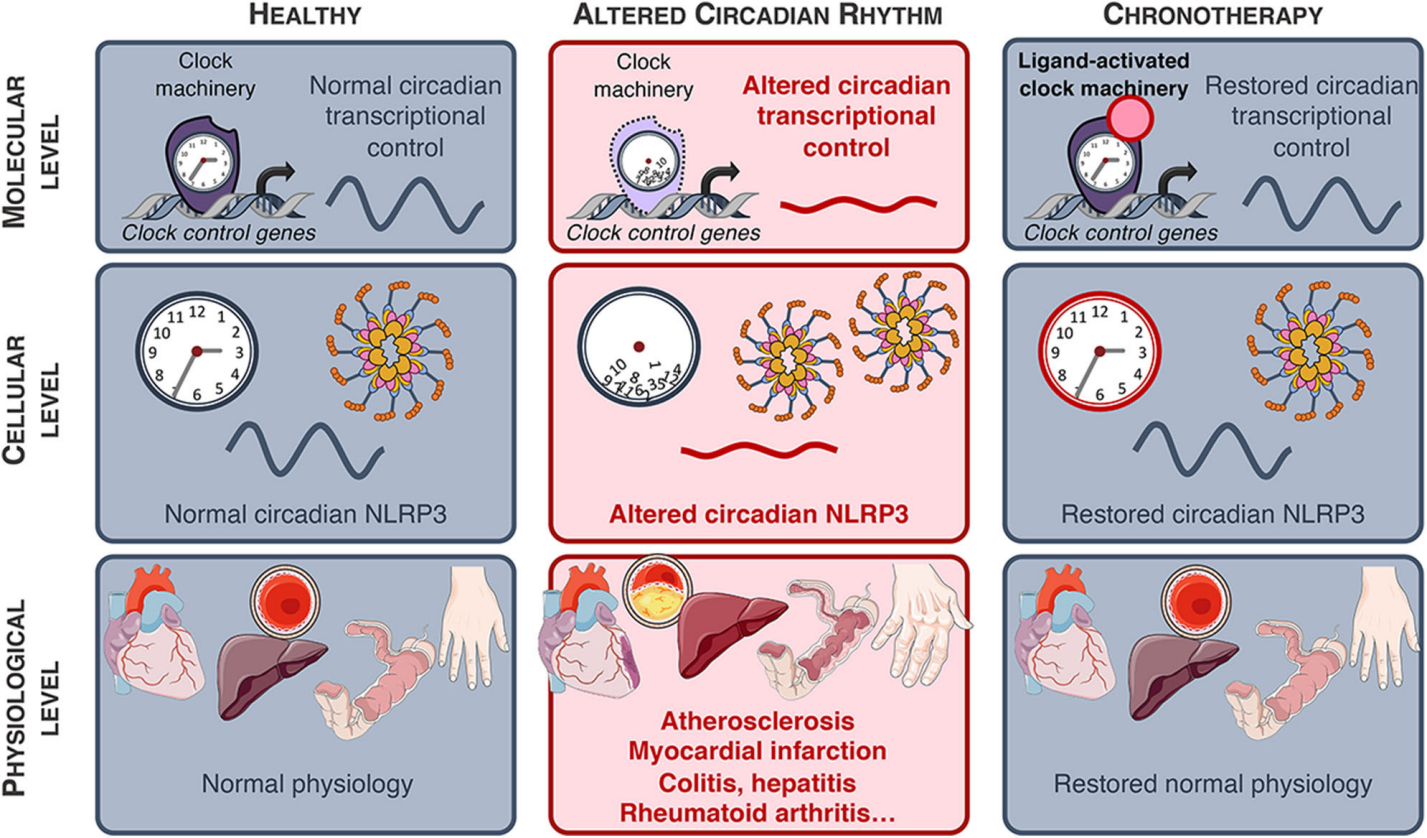
NLRP3-associated diseases. NLRP3-associated diseases and potential for innovative chronotherapies.

Virtually all mammalian cell types harbor a functional circadian clock, leading to circadian oscillations in the transcriptome, proteome, and ultimately cell/tissue function. The central pacemaker is located in the suprachiasmatic nucleus of the hypothalamus. It receives light information and synchronizes clocks throughout the body according to this time cue. The clock is present in immune cells including macrophages, lymphocytes and neutrophils as well as in lymphoid tissues such as the spleen and lymph nodes (40). The number of circulating leukocytes oscillates diurnally, peaking during the rest phase, due to circadian variations in haematopoietic cell egress from bone marrow which preferentially occurs at the onset of this phase (Figure 3) (41, 42). In addition, tissue leucocytes display circadian variations mainly due to oscillations in their rolling and adhesion to the endothelium and infiltration into tissues which predominantly occurs at the onset of the active phase (43, 44). In parallel, immune cell functions such as cytokines production, phagocytosis of exogenous particles or response to pathogens also display daily oscillations resulting in time-of-day-dependent difference in the susceptibility to septic shock or injury (45–48). This temporal organization is meant to ensure an optimization of the immune response in order to maintain or rapidly and efficiently restore homeostasis after infection or injury/tissue damage. Consequently, clock disruption has often been associated with inflammatory diseases (Figure 4).

## Circadian Control of the NLRP3 Inflammasome and Implication in Physiology and Pathologies

Expression of the NLRP3 inflammasome complex components is low and increased transcription is achieved during the priming step. It was recently demonstrated that the mRNA expression of the components of the NLRP3 inflammasome oscillates in a daily manner under the control of Rev-erbα in peritoneal mouse macrophages *in vivo*, reaching a peak (or zenith) during the active phase, corresponding to the lowest expression (or nadir) of Rev-erbα mRNA (21). Similar oscillations were observed in primary mouse bone marrow-derived and human monocyte-derived macrophages synchronized by a serum shock *ex vivo*, while circadian oscillations in *Nlrp3* mRNA were lost upon Rev-erbα ablation. NLRP3 protein amounts were accordingly regulated upon modulation of Rev-erbα activity (21). Strikingly, alteration in the NLRP3 pathway provoked by impairment of *Rev-erb*α expression triggers alterations in IL-1β and IL-18 secretion in peritoneum (21). Additionally, Rev-erbα also regulates the NLRP3 activation step. Indeed, *Rev-erb*α ablation led to increased speck formation, caspase-1 cleavage and NLRP3-induced caspase-1 mediated maturation and secretion of IL-1β and IL-18 in LPS-primed macrophages activated with nigericin or ATP. By contrast, activation of Rev-erb by its natural (heme) or pharmacological ligands reduced the secretion of these pro-inflammatory cytokines. Mechanistically, Rev-erbα binds to specific response elements in *Nlrp3* and *Il1*β gene promoters to silence their expression, and controls the NLRP3 inflammasome assembly and caspase 1 maturation (21) (Figure 1). In an *in vivo* model of acute sterile peritonitis induced by intraperitoneal administration of LPS and a concomitant injection of alum to specifically activate the NLRP3 pathway, IL-1β and IL-18 plasma levels were higher in *Rev-erb*α-deficient mice toward the end of the resting phase when Rev-erbα expression is highest in the wild-type controls, whereas the difference was lost during the active phase when Rev-erbα is nearly absent. Accordingly, and although this was not studied at different times of the day, Rev-erbα inhibition with an antagonist exacerbates the severity of LPS-induced acute lung injury by increasing NLRP3-dependent IL-1β secretion (49). These data demonstrate the rhythmic regulation of the NLRP3 inflammasome expression and activation, and suggest that Rev-erb pharmacological modulation may exert beneficial action in acute or chronic inflammatory diseases in which the NLRP3 inflammasome is over-activated, as detailed below.

### Fulminant Hepatitis

Fulminant hepatitis (FH) is a life-threatening condition characterized by a fast-evolving hepatic dysfunction associated with encephalopathy and coagulopathy (50, 51). Numerous factors such as viral infection, metabolic and genetic diseases as well as absorption of toxic compounds are able to trigger FH, although overdose of acetaminophen still remains the main cause of FH nowadays (52). Acetaminophen accumulation leads to the production of large quantities of toxic metabolites provoking oxidative stress, mitochondrial membrane potential loss and hepatocellular death, the secretion of DAMPs and activation of the NLRP3 inflammasome (52). Increased IL-1β and activation of the NLRP3 inflammasome in macrophages has also been shown in viral hepatitis (53–55). Strikingly, the susceptibility of FH is time-of-day dependent, upon the control of the molecular clock and Rev-erbα was identified as an important regulator of the inflammasome in this context. In mice, ablation of Rev-erbα led to exacerbated fulminant hepatitis, including increased liver damage that was blunted upon administration of the MCC950 specific NLRP3 inhibitor (Table 1) (21, 58). Remarkably, Rev-erbα pharmacological activation dramatically reduced liver injury thereby delaying death and improving the rate of survival from fulminant hepatitis from 10% in the control to 70% in the treated mice (Figure 4) (21).

**Table 1:** NLRP3-associated diseases linked to the clock.

**NLRP3-associated disease**	**Clock intervention with effects on NLRP3**	**References**
Atherosclerosis	Not tested	
Colitis	• Rev-erbα KO, Bmal1 KO: worsening • Jetlag: worsening • Rev-erbα agonist: improvement	(56)
Fulminant hepatitis	• Rev-erbα KO: worsening • Rev-erbα agonist (mice): improvement • Chrono-pharmacological approach (mice)	(21)
Gout	Not tested	
Lung injury	Rev-erbα antagonist: worsening Rev-erbα agonist: improvement	(49)
Myocardial infarction/ischemia-reperfusion injury & heart failure	• Rev-erbα KO (mice, *in vivo*): worsening • Rev-erbα agonist (mice, *in vivo*, at ZT6/ZT18): improvement • Chrono-pharmacological approach	(57)
NASH	Not tested	
Rheumatoid arthritis	Not tested	
Type 2 Diabetes	Not tested	

### Colitis

Several studies have suggested a role of the NLRP3 inflammasome in inflammatory intestinal diseases and although controversial results were first published, it is now accepted that NLRP3 activation is detrimental in this context. Ablating IL-18 or blocking its signaling reduced the severity of experimental colitis (59, 60). In addition, the NLRP3 inflammasome was identified as a central mediator of intestinal inflammation in dextran sulfate sodium (DSS)-induced colitis (61). Consistent with the previously described role of the clock in the regulation of the NLRP3 activation, DSS-induced colitis was found to be more severe in mouse models of environmental or genetic disruption of the clock (56). Confirming previous results, *Rev-erb*α-deficient mice were found to display increased activation of the NLRP3 pathway which accounted for the severe phenotype, whereas pharmacological Rev-erb activation attenuated colitis *in vivo* (Figure 4). Interestingly, when tested *in vitro*, the Rev-erb agonist seems to be active only on the priming step, and was ineffective at modifying caspase 1 maturation in cultured LPS-primed macrophages activated with ATP. This might be due to the fact that the cells were not synchronized in this study. Still, the effects of Rev-erb activation were abolished by MCC950, a specific inhibitor of the NLRP3 inflammasome activation.

### Cardio-Vascular Diseases

#### Circadian Clock and Blood Vessels Physiology

Circadian clocks reside in the different cell types of blood vessels (62, 63) and participate in vascular function and tone (64). For instance, blood pressure displays circadian oscillations, starting to rise before the rest-to-active transition while being lower during sleep (65), coinciding with the higher frequency of acute cardiovascular events and the exacerbated acute thrombus formation in the early morning hours (66, 67). Circadian oscillations in clock genes expression are attenuated in human atherosclerotic plaque (68), suggesting a mechanistic link between altered clock function and vascular pathologies. Numerous studies revealed that clock disruption (e.g., altered sleep patterns, shift-work) increases cardiovascular risk factors such as dyslipidemia, diabetes, hypertension and lead to cardiovascular diseases including stroke and coronary heart disease (Figure 4) (66, 69). Several studies found a relationship between shift work or acute circadian misalignment and subclinical atherosclerosis, measured by higher intima-media thickness (IMT) and elevated systemic inflammation even after adjustment for age and common risk factors (70–73). Moreover, lower sleep duration and fragmented sleep are independently associated with an increased risk of subclinical coronary and non-coronary atherosclerosis (74).

#### Clock Control of NLRP3 Inflammasome Activation, IL-1β Production and Atherogenesis

Atherosclerosis is a lipid-driven inflammatory disease of the arterial wall. Infiltration and modification of lipoproteins in the subendothelial space result in their uptake mainly by macrophages, forming foam cells, thus initiating atherosclerotic lesion formation. Then, lipids (fatty acids, ox-LDL, cholesterol crystals...) accumulate as well as inflammatory cells, notably monocyte-derived macrophages, T and B lymphocytes (75–77). Inefficient efferocytotic removal of these foam cells and apoptotic cells promote lesion progression toward advanced lesions with a necrotic core, degradation of the extracellular matrix, migration of smooth muscle cells and in some cases calcification, which may become vulnerable (78). Genetic alteration of the molecular clock contributes to metabolic imbalance and inflammation which promote atherogenesis (79, 80). For instance, BMAL1 modulates lipoprotein production and biliary cholesterol excretion, and its ablation led to hyperlipidemia and atherosclerosis (81). In the same line, Rev-erbα diminishes atherogenic lipoproteins plasma levels (82), modulates the inflammatory profile of macrophages toward an anti-inflammatory phenotype (83) while its activation reduced atherogenesis (84). Accordingly, BMAL1 regulates macrophage polarization as well as the cyclic trafficking of Ly6C^hi^ monocytes and myeloid *Bmal1* deletion increased monocyte recruitment and worsened atherosclerosis (Figures 2, 3) (85). Pro-inflammatory recruitment through the CCL2 (MCP-1)-CCR2 axis plays an important role in plaque development (86). In a recent study, McAlpine and colleagues revealed that sleep modulates haematopoiesis while chronic sleep fragmentation in a mouse model prone to atherosclerosis resulted in increased production of Ly6C^high^ monocytes and aggravated atherosclerosis development due to increased infiltration to the lesions (Figure 4) (87). In line, disruption of circadian rhythms by chronic jetlag obtained by weekly alternating light-dark cycles with 12 h shifts enhanced atherosclerosis development and increased lesion macrophage content (Figure 4) (88). Interestingly, Winter et al. elegantly showed that myeloid cells are recruited to the lesions in a circadian manner, with a peak during the active-to-rest transition, through the rhythmic deposit of CCL2 on the arterial endothelium by circulating cells. A chrono-pharmacological approach targeting monocyte recruitment *via* timed inhibition of the CCR2/CCL2 axis during the active phase dampened atherosclerotic lesions development (Figure 4) (89).

In atherosclerotic lesions, oxLDL can prime the macrophage NLRP3 inflammasome by activating TLRs-dependent pathways. In addition, CD36-mediated oxLDL uptake eventually results into intra-lysosomal crystallization. Together with phagocytized extracellular cholesterol, they are thought to trigger macrophage lysosomal damage thus provoking cathepsins release (18). Moreover, defective cholesterol efflux in myeloid cells results in accumulation of unesterified cholesterol which contributes to both priming and activation of the NLRP3 inflammasome, promoting neutrophil recruitment and neutrophil extracellular trap (NET) formation in atherosclerotic plaques (90). The NLRP3 inflammasome activation contributes to the vascular inflammatory response through enhanced production of IL-1α and IL-1β, the latter driving inflammation during early atherogenesis and the evolution of advanced atheroma in mice (91). Canakinumab is an IL-1β-neutralizing antibody approved for the treatment for CAPS-associated symptoms which also reduced the incidence of two other NLRP3-related diseases, arthritis and gout (92). Recently, the CANTOS (Canakinumab Anti-inflammatory Thrombosis Outcome Study) study demonstrated that IL-1β neutralization decreased the incidence of atherosclerotic disease and reduced systemic inflammation in at-risk patients with previous myocardial infarction in the absence of effect on lipids, indicating that suppressing IL-1β contributes to the reduction in cardiovascular risk (93). However, substantial residual inflammatory risk still subsisted after IL-1β neutralization, with on-treatment IL-18 and IL-6 plasma levels associated with future cardiovascular risk (94), advocating for therapies that simultaneously inhibit IL-1β and IL-18. In mice, inhibition of the NLRP3 inflammasome reduces atherogenesis in *ApoE*^−/−^ or *LDLr*^−/−^mice (24, 95). Although plausible, it is currently unclear whether circadian control of NLRP3 inflammasome activation is perturbed within macrophage foam cells from atherosclerotic lesions. In this perspective, a therapy that targets the clock, and particularly Rev-erbα, in a chrono-pharmacological approach would be worth testing as Rev-erbα not only regulates NLRP3 inflammasome expression and activation, reducing both IL-1β and IL-18, but also MCP-1 expression and IL-6 production by macrophages (Figure 4) (47, 96), as well as lipoprotein metabolism, thereby simultaneously impacting both local inflammation and systemic risk factors.

### Myocardial Infarction and the Circadian Control of NLRP3 Expression and Activation

#### The NLRP3 Inflammasome Is Activated Upon Acute Myocardial Ischemia/Reperfusion Injury

Myocardial infarction (MI) is one of the leading causes of death worldwide and is associated with a poor quality of life, acknowledging the increased interest in finding novel therapeutics to reduce reperfusion injury and preserve cardiac function. Despite improvement in reperfusion and treatment strategies that have led to higher survival rates, fibrosis and adverse left ventricular remodeling consecutive to reperfusion injury leads to cardiac contractile dysfunction and eventually heart failure (97).

Acute myocardial infarction (AMI) initiates a sterile inflammatory response that enables necrotic cardiomyocyte debris removal, angiogenesis and wound healing (98); however, this inflammatory response also promotes cell death by pyroptosis, expanding infarct size, and results in fibrosis and adverse ventricular remodeling. Then, refined intervention to rapidly attenuate this inflammatory burst is desirable (99). IL-1β and IL-18 are rapidly increased upon MI. Interestingly, administration of IL-1β- or IL-18-neutralizing antibody inhibits cardiomyocyte apoptosis, reduces infarct size and improves cardiac dysfunction after MI in mice (100, 101). In line, reduction of IL-1β production in caspase1- and in ASC-deficient mice upon ischemia/reperfusion is associated with a marked reduction in the infarct size, left ventricle remodeling and myocardial fibrosis (102). These data suggested that activation of the inflammasome may provoke further tissue damage through caspase-1-mediated production and release of IL-1β. Sandanger et al. confirmed that *Nlrp3* deletion in mice leads to reduced infarct size and preservation of cardiac function in isolated perfused hearts subjected to acute I/R *ex vivo* (103).

Expression of the NLRP3 inflammasome components is very low and priming is induced during ischemic injury by cellular debris. NLRP3 is then activated by extracellular ATP as well as cardiolipin and mtDNA released by dying cells from damaged tissue after acute ischemic injury, or within minutes of reperfusion due to sudden surge of reoxygenation-induced ROS production and mitochondrial damage (Figure 1) (98). The NLRP3 inflammasome also senses extracellular-mediated efflux of K^+^ in cardiac fibroblasts upon hypoxia (103). In addition, the NLRP3 inflammasome is activated by numerous danger signals stemming from co-morbidities such as high glucose and lipid levels and derivatives (ceramides, advanced glycation products, which may lead to chronic activation of the NLRP3 inflammasome locally or in other organs) (104).

#### Inhibition of the NLRP3 Activation as a Novel Strategy to Reduce Myocardial I/R Injury?

Strategies to inhibit the activation of NLRP3 in the early reperfusion period after ischemic MI to reduce infarct size, avoid adverse remodeling and fibrosis and ameliorate cardiac function have been tested. Several inhibitors of the NLRP3 inflammasome activation have been developed, enabling pharmacological intervention in animal models undergoing AMI. Administration of the NLRP3 inhibitor MCC950 (58) lowers infarct size and area at risk (105, 106). Remarkably, NLRP3 inhibition by MCC950 treatment was associated with preserved left ventricle (LV) ejection fraction (LVEF), reduced fibrosis and myocardial immune cell infiltration. Interestingly however, the benefit of administering the NLRP3 inhibitor before AMI or within 1 h of reperfusion was lost when the NLRP3 inhibitor was given after 3 h of reperfusion, suggesting that inhibition should be achieved at time of NLRP3 assembly and activation (107). Other NLRP3 inhibitors have been shown to reduce infarct size in mouse models of myocardial ischemia/reperfusion. Among them, OLT1177 reduces infarct size in mice (108) and is currently in Phase 1b in a randomized, double-blinded, placebo-controlled, safety, and pharmacodynamics study in 30 subjects with stable systolic heart failure (HF) with impaired LVEF.

#### Circadian Rhythms in Cardiac Biology and Diseases

Daily oscillations of blood pressure and heart rate are reduced or lost in cardiomyocyte-specific Clock-mutant (109) acknowledging the important role of cardiomyocyte clock machinery in cardiac function. Consistently, circadian disruption due to either environmental out-of-sync stimuli or genetic manipulation of clock genes results in cardiomyopathies, cardiac dysfunction, arrhythmia, and reduced survival (110–113) and for review (114) (Figure 4). Additionally, circadian variations are seen in the onset and frequency of myocardial infarction, stroke and sudden death (115, 116), as well as in the severity of the diseases (117). Furthermore, environmental circadian disruption adversely impacts cardiac remodeling and function, increases macrophage infiltration and led to cardiac hypertrophy in mice undergoing MI (Figure 4) (118). The circadian influence in the tolerance to I/R injury was corroborated in mice undergoing I/R at the resting-to-active and active-to-rest transitions. The former led to exacerbated infarct size, and subsequent fibrosis and adverse cardiac remodeling. This time-of-day difference in the tolerance to I/R was markedly attenuated in cardiomyocyte-specific circadian clock mutant mice (119). In humans, it was recently assessed whether myocardial tolerance of I/R differed depending on the timing of aortic valve replacement surgery, as measured by the occurrence of major adverse cardiovascular events (cardiovascular death, myocardial infarction, and admission to hospital for acute heart failure). Expectedly, perioperative myocardial injury was better tolerated when patients underwent surgery in the afternoon (120). Interestingly, targeting the circadian clock through pharmacological modulation of Rev-erbα/β in mice was able to reduce myocardial I/R injury *ex vivo* in an isolated Langendorff-perfused mouse heart model of hypoxia-reperfusion, thus providing new therapeutic ways to dampen the adverse outcome of cardiac I/R injury. Whether Rev-erbα might be an interesting target to reduce cardiac dysfunction was further established in a model of transaortic constriction-induced heart failure (121) as well as in a mouse model of AMI (122). In this later report, the author suggested that blunted inflammation and reduced recruitment of neutrophils and pro-inflammatory macrophages upon pharmacological Rev-erbα modulation may, at least in part, contribute to the benefit of targeting Rev-erbα. Remarkably, Schloss et al. elegantly demonstrated that infarct size is higher at ZT13 vs. ZT5 when the number of cardiac Ly6C^high^ monocytes is highest likely because of increased CCR2-mediated recruitment of these cells, and that blocking the CCR2-CCL2 axis blunted the time-of-day variations in infarct size (123). These data, together with the observation that Rev-erbα controls macrophage NLRP3 activation (21), point to a possible role of monocyte/macrophage Rev-erbα in I/R tolerance. In a recent report, Martino and colleagues questioned the cell-specific role of Rev-erbα in the protective effect of Rev-erbα activation. They found that activating Rev-erbα at time of reperfusion in wild-type mice limits infarct expansion, improves cardiac function and outcomes, and reduced recruitment of neutrophils and macrophages as well as cardiac NLRP3 inflammasome activation. However, myeloid cells were unlikely to account for this beneficial effect as shown by bone marrow transfer experiments (57). Instead, Rev-erbα may downregulate the NLRP3 inflammasome in cardiac fibroblasts although further studies using cell-specific mutant mice are necessary to pinpoint the exact contribution of each cell types. More importantly, pharmacological Rev-erbα activation showed the greatest benefit when given at time of reperfusion, whenever it happened during the active (ZT18) or resting (ZT6) phase, although the benefit was greater at ZT6 corresponding to maximal Rev-erbα expression (Figure 4). This suggests that beyond the time of treatment and potentially differential effect on the pace of the clock, Rev-erbα-regulated inhibition of NLRP3 before or at reperfusion may hold promise to reduce myocardial I/R damages, whenever the time at which the patient will undergo cardiac surgery or experience MI.

## Conclusion

In this review, we have highlighted the relationship between circadian immunity and the NLRP3 inflammasome pathway. As a central sensor of tissue damages and metabolic imbalance, NLRP3 plays a pivotal role in tissue homeostasis in many tissues including liver, heart and the vasculature. Sustained activation of NLRP3 by exogenous or endogenous triggers thus aggravates chronic inflammatory diseases such as atherosclerosis (24) or worsen acute inflammatory conditions such as fulminant hepatitis (21) or myocardial infarction (121). As such, the NLRP3 inflammasome represents an innovative target, as exemplified by the use of NLRP3 inhibitors in several disease models. However, MCC950 displays hepatotoxic properties, advocating for the development of alternative NLRP3 inhibitory strategy (124). Strikingly, because NLRP3 is controlled by the clock machinery, the time of exposure to intruders and their sensing has a dramatic impact on the inflammatory response amplitude, the disease outcome and its resolution. As such, a chrono-pharmacological approach targeting NLRP3 may have greater benefits for the treatment of NLRP3-driven diseases (Figure 4). Since pathological tissues often display distinct circadian oscillation patterns compared to healthy tissue, such strategies would allow to target NLRP3 specifically in pathological areas and then preserve homeostasis in healthy tissue and thus reduce adverse effects. Several targets should be considered, either the NLRP3 pathway itself or NLRP3-regulating clock components such as Rev-erbα. Finally, alteration of NLRP3 pathway is involved in other diseases including diabetes, Alzheimer disease, gout, rheumatoid arthritis, or asthma (5). Clock-driven NLRP3 resynchronisation may represent an additional approach to help treating these diseases.

## Author Contributions

All authors listed have made a substantial, direct and intellectual contribution to the work, and approved it for publication.

## Conflict of Interest

The authors declare that the research was conducted in the absence of any commercial or financial relationships that could be construed as a potential conflict of interest.
